# Supplementary material for: FERN – a Java framework for stochastic simulation and evaluation of reaction networks
Source: BMC Bioinformatics. 2008 Aug 29;9:356. doi: 10.1186/1471-2105-9-356 (PMC2553347; doi:10.1186/1471-2105-9-356)
Supplement: Additional file 1 — FERN distribution, Version 1.3. This archive contains the FERN source code and binaries as well as documentation and example models in FernML and SBML. [file 1471-2105-9-356-S1.zip › fern/doc/javadoc/fern/tools/gnuplot/ArrayMatrixAxes.html]

ArrayMatrixAxes


---


|  |  |  |
| --- | --- | --- |
| **Overview** \| **Package** \| **Class** \| **Use** \| **Tree** \| **Deprecated** \| **Index** \| **Help** | |  |
| PREV CLASS   **NEXT CLASS** | **FRAMES**    **NO FRAMES**     **All Classes** |  |
| SUMMARY: NESTED \| FIELD \| CONSTR \| METHOD | DETAIL: FIELD \| CONSTR \| METHOD |  |


---


## fern.tools.gnuplot Class ArrayMatrixAxes

```
java.lang.Object
  fern.tools.gnuplot.Axes
      fern.tools.gnuplot.ArrayMatrixAxes
```

**All Implemented Interfaces:**: Iterable<String>

**Direct Known Subclasses:**: TransposedArrayMatrixAxes

---

``` public class ArrayMatrixAxes extends Axes ```

Implementation of `Axes` to use with array matrixes
of any number type (the type of the arrays is inferred by
using the reflection api). An array matrix is just a two dimensional
rectangular array (`new double[3][20]`). The first index
of the matrix gives the row, the second the column (3 rows and 20 columns).

If your array matrix is transposed (your first index denotes the column, the second
one the row), just use `TransposedArrayMatrixAxes`.

**Author:**
:   Florian Erhard

**See Also:**: `Axes`

---

| **Field Summary** | |
| --- | --- |
| `protected  Object` | `array`             Contains the matrix array |


**Constructor Summary**

`ArrayMatrixAxes(Object matrix)`
  
          Create a new `Axes` object containing the given array matrix
without labels / styles.

`ArrayMatrixAxes(Object matrix,
String[] labels,
String[] styles)`
  
          Create a new `Axes` object containing the given array matrix
with the given labels /styles (either can be `null`).


| **Method Summary** | |
| --- | --- |
| `protected  void` | `checkMatrix()`             Checks if the passed object is a valid array matrix. |
| `int` | `getNumColumns()`             Gets the number of columns. |
| `int` | `getNumRows()`             Gets the number of rows. |
| `Iterator<String>` | `iterator()`             Yields the tab separated columns row by row. |

**Methods inherited from class fern.tools.gnuplot.Axes**

`addAxes, applyDefaultStyle, getAdditionalAxesIterator, getLabel, getNumAdditionalColumns, getStyle, loadLabelsAndLineStyles, setLabel, setStyle`

**Methods inherited from class java.lang.Object**

`clone, equals, finalize, getClass, hashCode, notify, notifyAll, toString, wait, wait, wait`

**Field Detail**

### array

```
protected Object array
```

:   Contains the matrix array


**Constructor Detail**

### ArrayMatrixAxes

```
public ArrayMatrixAxes(Object matrix)
```

:   Create a new `Axes` object containing the given array matrix
    without labels / styles. If the passed object is not a valid array matrix,
    an `IllegalArgumentException` will be thrown.

    **Parameters:**: `matrix` - array matrix

---


### ArrayMatrixAxes

```
public ArrayMatrixAxes(Object matrix,
                       String[] labels,
                       String[] styles)
```

:   Create a new `Axes` object containing the given array matrix
    with the given labels /styles (either can be `null`).
    If the passed object is not a valid array matrix,
    an `IllegalArgumentException` will be thrown.

    **Parameters:**: `matrix` - the array matrix: `labels` - the labels: `styles` - the styles


**Method Detail**

### checkMatrix

```
protected void checkMatrix()
```

:   Checks if the passed object is a valid array matrix.

---


### getNumColumns

```
public int getNumColumns()
```

:   **Description copied from class: `Axes`**
:   Gets the number of columns. The number of the attached `Axes`
    object is also included.

    :   **Specified by:**: `getNumColumns` in class `Axes`
    :   **Returns:**: number of columns.

---


### getNumRows

```
public int getNumRows()
```

:   **Description copied from class: `Axes`**
:   Gets the number of rows.

    :   **Specified by:**: `getNumRows` in class `Axes`
    :   **Returns:**: number of rows.

---


### iterator

```
public Iterator<String> iterator()
```

:   Yields the tab separated columns row by row.


---


|  |  |  |
| --- | --- | --- |
| **Overview** \| **Package** \| **Class** \| **Use** \| **Tree** \| **Deprecated** \| **Index** \| **Help** | |  |
| PREV CLASS   **NEXT CLASS** | **FRAMES**    **NO FRAMES**     **All Classes** |  |
| SUMMARY: NESTED \| FIELD \| CONSTR \| METHOD | DETAIL: FIELD \| CONSTR \| METHOD |  |


---
